# Host specificity and microhabitat preference of symbiotic copepods (Cyclopoida: Clausiididae) associated with ghost shrimps (Decapoda: Callichiridae, Callianideidae)

**DOI:** 10.1002/ece3.6726

**Published:** 2020-09-03

**Authors:** Vahid Sepahvand, Bryan L. Brown, Ali Gholamifard

**Affiliations:** ^1^ Department of Marine Biological Sciences Iranian National Institute for Oceanography and Atmospheric Science (INIOAS) Tehran Iran; ^2^ Department of Biological Sciences Virginia Tech Blacksburg VA USA; ^3^ Department of Biology Faculty of Sciences Lorestan University Khorramabad Iran

**Keywords:** *Callianidea*, *Clausidium*, *Neocallichirus*, Persian Gulf

## Abstract

We examined the host specificity of two ectosymbiotic *Clausidium* Kossman, 1874 copepods **(**Cyclopoida: Clausiididae**)** on two co‐occurrence species of host ghost shrimps. Our results revealed that both species of symbiotic copepod demonstrated extremely high host specificity. Moreover, within a single host shrimp species, each symbiont species displayed strong spatial patterns in microhabitat selection on their hosts’ bodies. *Clausidium persiaensis* Sepahvand & Kihara, 2017, was only found on the host *Callianidea typa* Milne Edwards, 1837 and almost exclusively within the host shrimp gill chamber, while *C. iranensis* Sepahvand, Kihara, & Boxshall, 2019 was only found on the host *Neocallichirus jousseaumei* (Nobili, 1904) and showed extremely strong preferences for the chelae and anterior walking legs. We also found that while the number of symbionts tends to increase with the host size, the two host species differed in the degree of symbiont infestation, with large *C. typa* hosting approximately 7× as many symbionts as the similarly sized *N. jousseaumeia*. The mechanisms resulting in the observed differences in infestation levels and microhabitat preferences of clausidium copepods among their hosts, including differences in physiology, burrowing pattern, and host grooming behavior should be further investigated.

## Graphical abstract

We examined the host specificity of two ectosymbiotic Clausidium copepods on two sympatric species of host ghost shrimps. Our results revealed that both species of symbiotic copepod demonstrated extremely high host specificity.

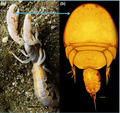

## 1 INTRODUCTION

Recent studies have revealed the ubiquitous nature of symbiotic relationships. While the majority of symbiotic relationships involve associations between organisms of disparate size, usually a larger host with a smaller symbiont, the degree of host specificity found among symbionts is remarkably variable. Some symbionts display extremely high plasticity in host selection, while other symbionts show perfect fidelity to a host species (Guo, Hwang, & Fautin, 1996; Ramirez, 1970) or even show preference for specific individuals within a species (Mills & Reynolds, 2002).

Habitat specificity—the selection of a particular domain on or inside the host's body—is also extremely common (Smyth & Halton, 1983). For example, monogenean gill parasites almost exclusively occur on the gills of their fish hosts and may even restrict their distributions to precise locations on those gills, including specific gill arches or a single side of the gills (Bychowsky, 1961; Rohde, 1979). While there are potentially numerous reasons for this type of site specificity, the specific physicochemical microhabitat is the most commonly invoked explanation for this phenomenon (e.g., Bychowsky, 1961; Wootten, 1974). Despite the ubiquity of this phenomenon, the mechanisms underlying host specificity are largely understudied.

From a symbiont's perspective, a population of potential hosts is a heterogeneous landscape. Hosts frequently vary in quality across species (Brown & Creed, 2004; Farrell, Creed, & Brown, 2014; Rohde, 1994) and there may even be significant variation in habitat quality across individuals within the same host species (Lie, 1973). Even at the within‐host level, microhabitats or specific tissues may vary with respect to the resources they offer, or the risk of mortality within each microhabitat patch (Mestre, Mesquita‐Joanes, Proctor, & Monrós, 2011; Skelton, Creed, & Brown, 2014; Skelton, Geyer, Lennon, Creed, & Brown, 2017). Moreover, each host and each microhabitat presents a limited pool of resources, creating the possibility of strong inter‐ and intra‐specific interactions among symbionts (Baker, Andras, Jordán‐Garza, & Fogel, 2013; Råberg et al., 2006; Ulrich & Schmid‐Hempel, 2012). Recently, Ivanenko et al. (2018) revealed that there was a lack of host specificity of associated copepods with mushroom corals in the red sea. The authors suggested that the association between copepods and their host corals is not strict, and not phylogenetically constrained. To address interaction among symbionts in one specific system, we investigated the host specificity and microhabitat preferences of two cyclopoid copepod associate with ghost shrimps.

Most clausidiid copepods live attached to the marine invertebrates host, and species of *Clausidium* Kossman, 1874 are known to live close association with burrowing shrimps (Boxshall & Halsey, 2004). The information on the behavior of these copepods, on their interactions with their host, with the environment, is very scarce.

Ghost shrimps (Decapoda: Axiidea) comprise decapod crustaceans that are adapted to a burrowing lifestyle (Poore, 1994). The burrows of ghost shrimp may house several species of symbionts. These symbionts are generally thought to be either parasitic or commensal, and include a variety of organisms, such as copepods (Jackson, 1996). The ghost shrimps *Callianidea typa* Milne Edwards, 1837 and *Neocalllichirus jousseaumei* (Nobili, 1904) are well distributed in the Persian Gulf and have been reported as hosts of cyclopoid copepods of the genus *Clausidium* (Sepahvand, Kihara, & Boxshall, 2019; Sepahvand, Rastegar‐Pouyani, Kihara, & Momtazi, 2017).

Marin and Nascimento (1993) analyzed the body size and habitat of *Callichirus garthi* (Retamal, 1975) (Decapoda: Callichiridae) as the factors affecting the distribution, abundance, and fecundity of their symbiotic copepods (*Clausidium* spp.). They (Marin & Nascimento, 1993) suggested that the density of *Clausidium* spp. per host was dependent upon host habitat type and recruitment. Corsetti and Strasser (2003) examined host selection of *Clausidium dissimile* Wilson, 1921 in two co‐occurrence populations of ghost shrimps. They found that, host‐size adjusted density of *C. dissimile* was affected by the host species and the months sampled. To test the hypothesis that particular copepod symbionts preferred particular locations on the host's body, we assessed microhabitat selection and host specificity of *Clausidium persiaensis* Sepahvand & Kihara, 2017 and *C. iranensis* Sepahvand et al., 2019 on the ghost shrimps *Callianidea typa* and *Neocallichirus jousseaumei*.

## 2 MATERIAL AND METHODS

**FIGURE 1:**
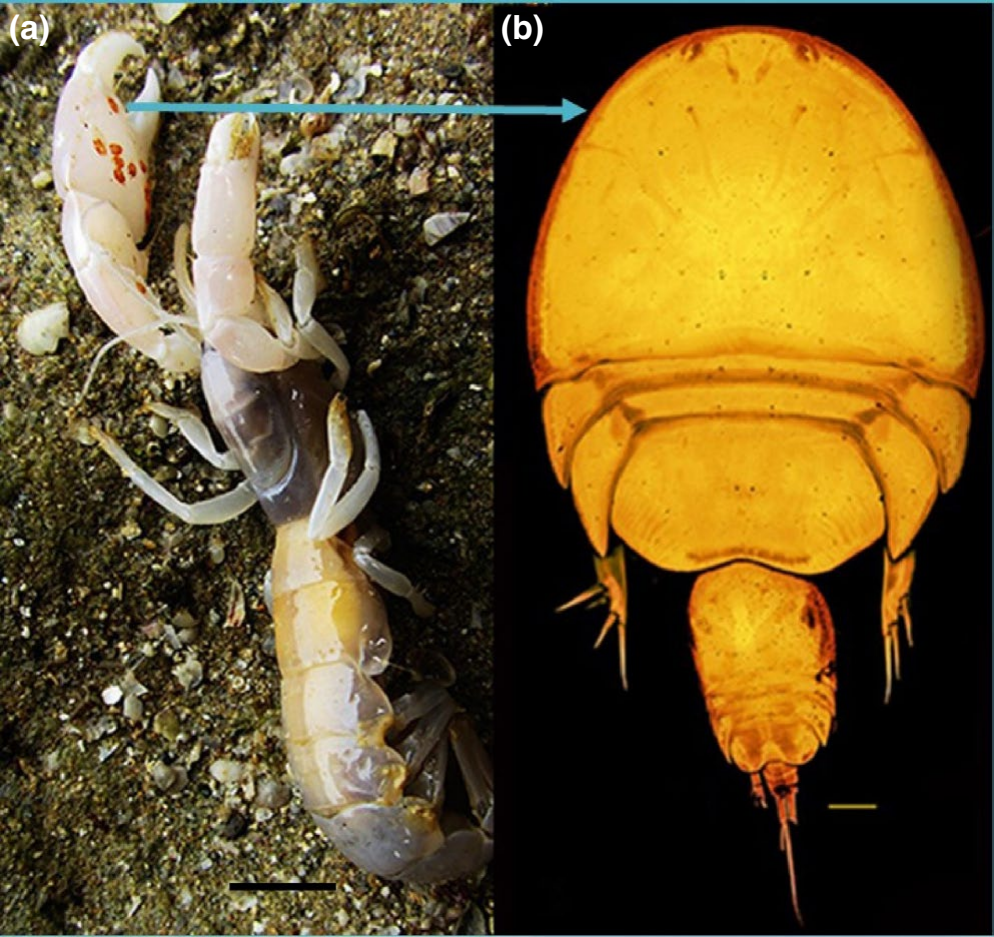
*Neocallichirus jousseaumei*: (A) habitus, cheliped with attached clausidiids; *Clausidium iranensis*. Scale bar: 1cm. (b) *Clausidium iranensis*. Confocal laser scanning microscopy maximum projections. Couple. Scale bar: 100 μm

**FIGURE 2:**
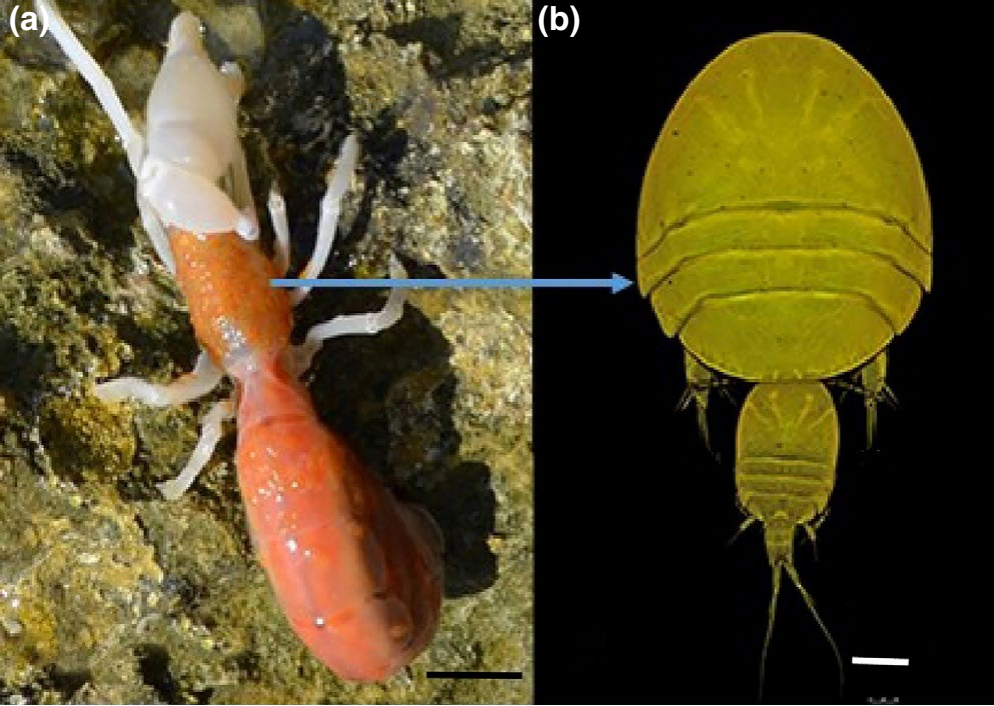
*Callianidea typa*: (a) habitus, carapace, and gill chamber with attached clausidiids; *Clausidium persiaensis*. Scale bar: 1 cm. (b) *Clausidium persiaensis*. Confocal laser scanning microscopy maximum projections. Couple. Scale bar: 100 μm

We conducted a field survey to collect ghost shrimps and their associated *Clausidium* copepods to assess host specificity, microhabitat selection, and relationships between symbiont abundance to host size. All organisms included in this study were collected at the Oli Village located in the coast of the Persian Gulf (27°50′14.62″N and 51°53′24.85″E). The ghost shrimp fauna at the site was dominated by *Neocallichirus jousseaumei* and *Callianidea typa* Milne Edwards, 1837 that supported the populations of the symbiotic copepods *Clausidium iranensis* Sepahvand et al., 2019 (Figure 1) and *Clausidium persiaensis* Sepahvand and Kiahara, 2017 (Figure 2). A lever was used for lifting boulders or splitting layered rocks to find the exposed host specimens. We collected 224 specimens of *Neocallichirus jousseaumei* and 125 specimens of *Callianidea typa* from June 2016 to December 2016.

Total length (TL, measured from the tip of the rostrum to the posterior end of the telson) and carapace length (CL, measured from the tip of the rostrum to the posterior end of the carapace) were recorded for each ghost shrimp. We identified ghost shrimps to species and transported each species to the laboratory in separate collection falcon tube to prevent interspecific transfer of clausidium copepods. We collected copepod symbionts from submerged hosts in the laboratory using a dissecting microscope.

**FIGURE 3:**
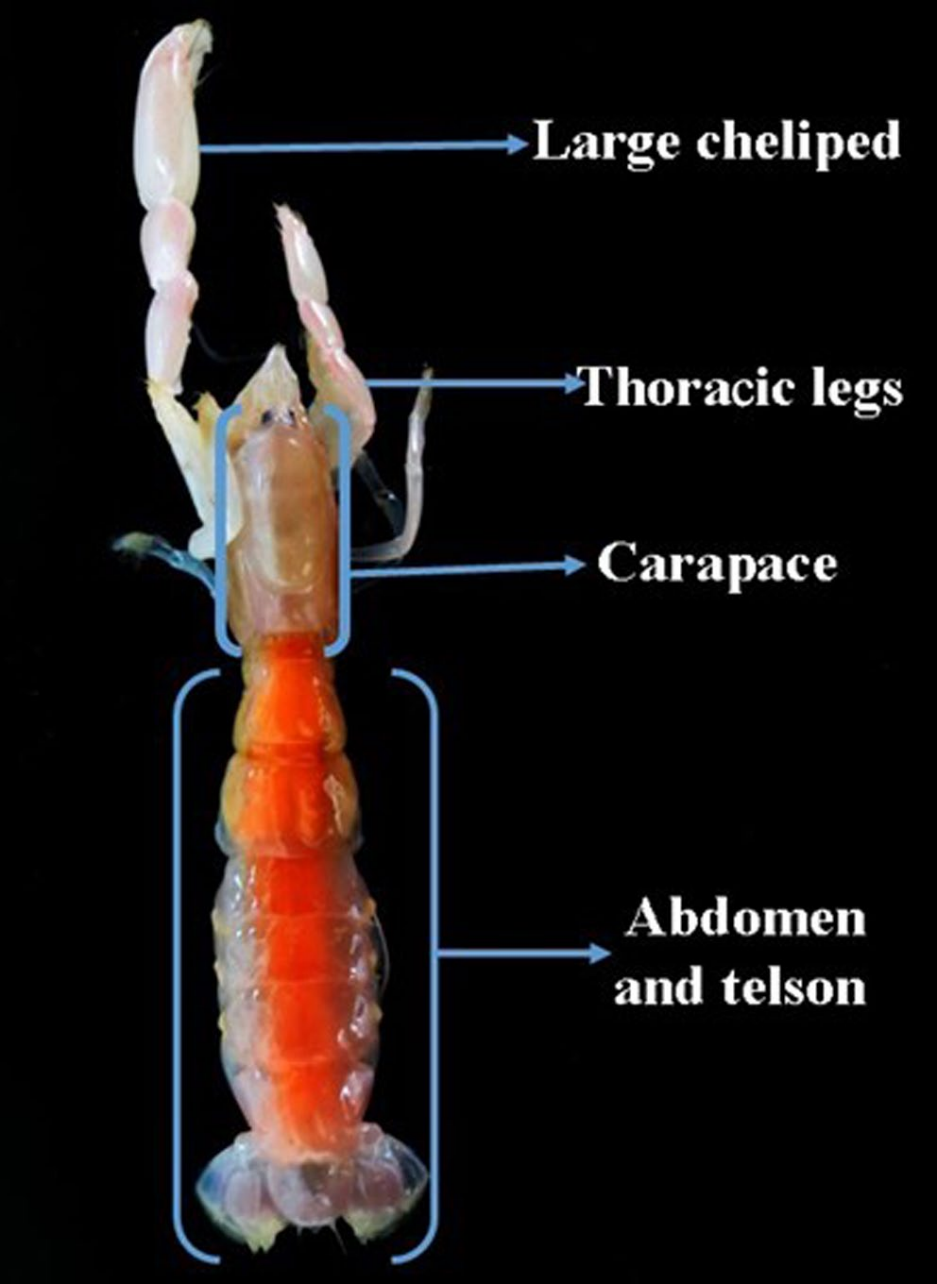
The four most commonly occupied microhabitats observed during field study

In order to map the distribution of copepods on their host, we divided the exoskeleton of the ghost shrimp into four regions based on natural morphological divisions, as illustrated in (Figure 3). We removed copepods by hand and recorded the location of each copepods on the exoskeleton.

We analyzed the distribution and intensity of symbiotic copepods using multiple methods that allowed us examine both the predictors of host intensity, as well as the multivariate distributions of symbiont locations on hosts. All analyses were performed with the statistical software R (R Core Team). We used a general linear mixed model of symbiotic copepod intensity on locations of each host using the function lme ( ) in the R package nlme. Host size and host sex were predictors in all models, and for models of nondominant sites, we included intensity at the dominant site as an additional predictor where dominant sites were defined as the site on each host with the highest intensity. This additional model factor allowed us to examine whether intensity at nondominant sites was related to intensity at dominant sites. When this term in the model was significant, we subsequently examined the relationship in intensity between sites using correlations of relative proportions. Relative proportion was used to correct for differences in total abundances across hosts. We examined the multivariate distribution of symbionts on hosts by including intensities at all sites in a nonmetric multidimensional scaling (NMDS; function metaMDS ( ) in the R package vegan) and permutational Analysis of Variance (PERMANOVA; function Adonis ( ) in the R package vegan). For all multivariate analyses, we used Euclidean distance. While Bray‐Curtis is often employed as a distance metric when analyzing community data, in our case, the copepod symbiont community was censused on each host rather than sampled, so observations of zero at a site are meaningful and Euclidean distance was more appropriate. We also examined the multivariate dispersion of symbionts using permutational analysis of dispersion (Anderson, 2006; function betadisper ( ) in the R package vegan).

## 3 RESULTS

**FIGURE 4:**
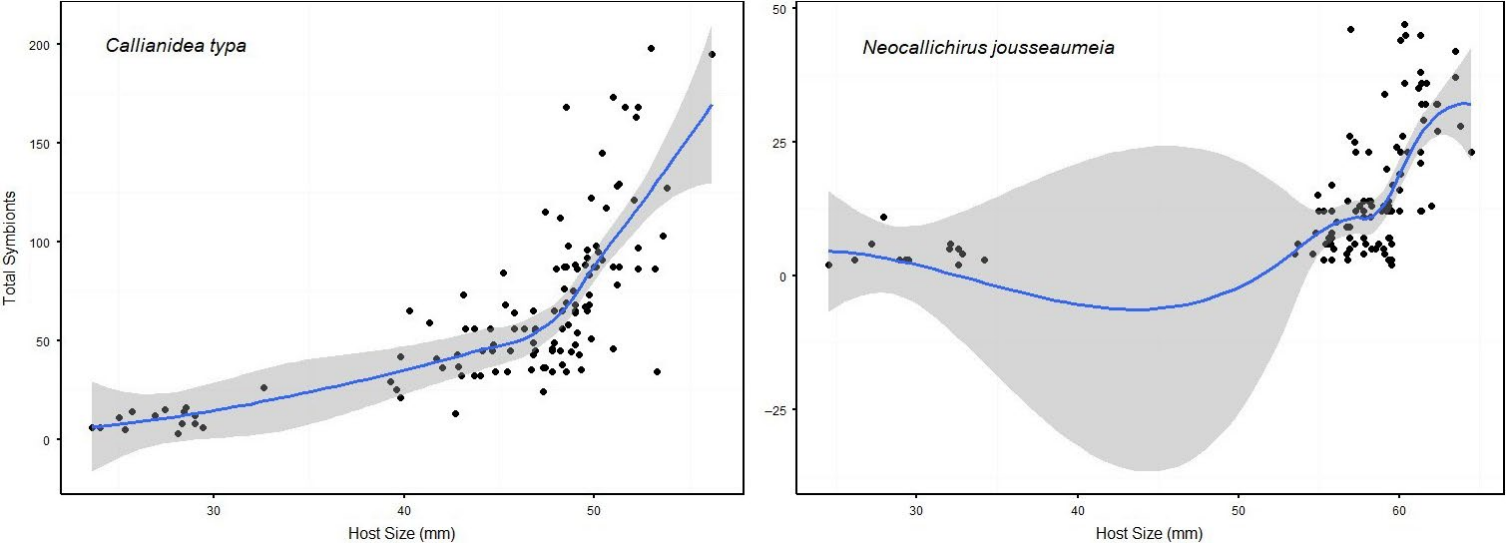
Relationships between total number of copepod symbionts per host and host size for the shrimp hosts *Callianidea typa* and *Neocallichirus jousseaumeia*. Solid lines represent a 1st order spline fit to the data with shaded areas indicating 95% confidence intervals

**FIGURE 5:**
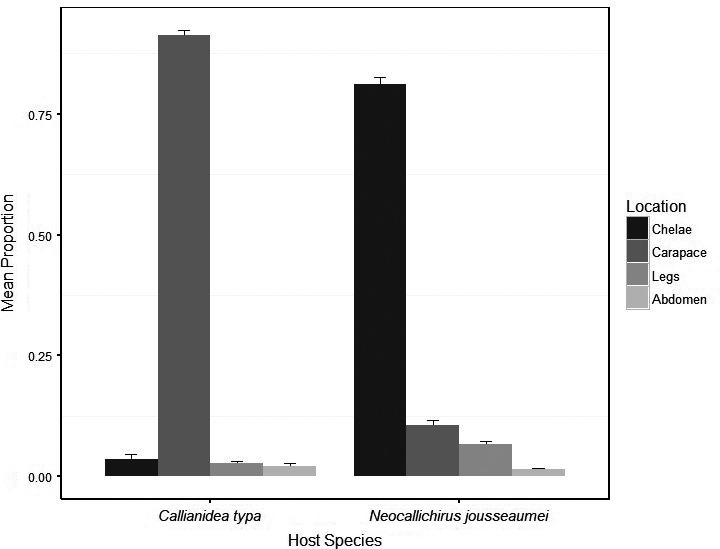
Proportion of copepod symbionts that occupied each of 4 host body regions on the shrimp hosts *Callianidea typa* and *Neocallichirus jousseaumeia*. Error bars represent standard errors

**FIGURE 6:**
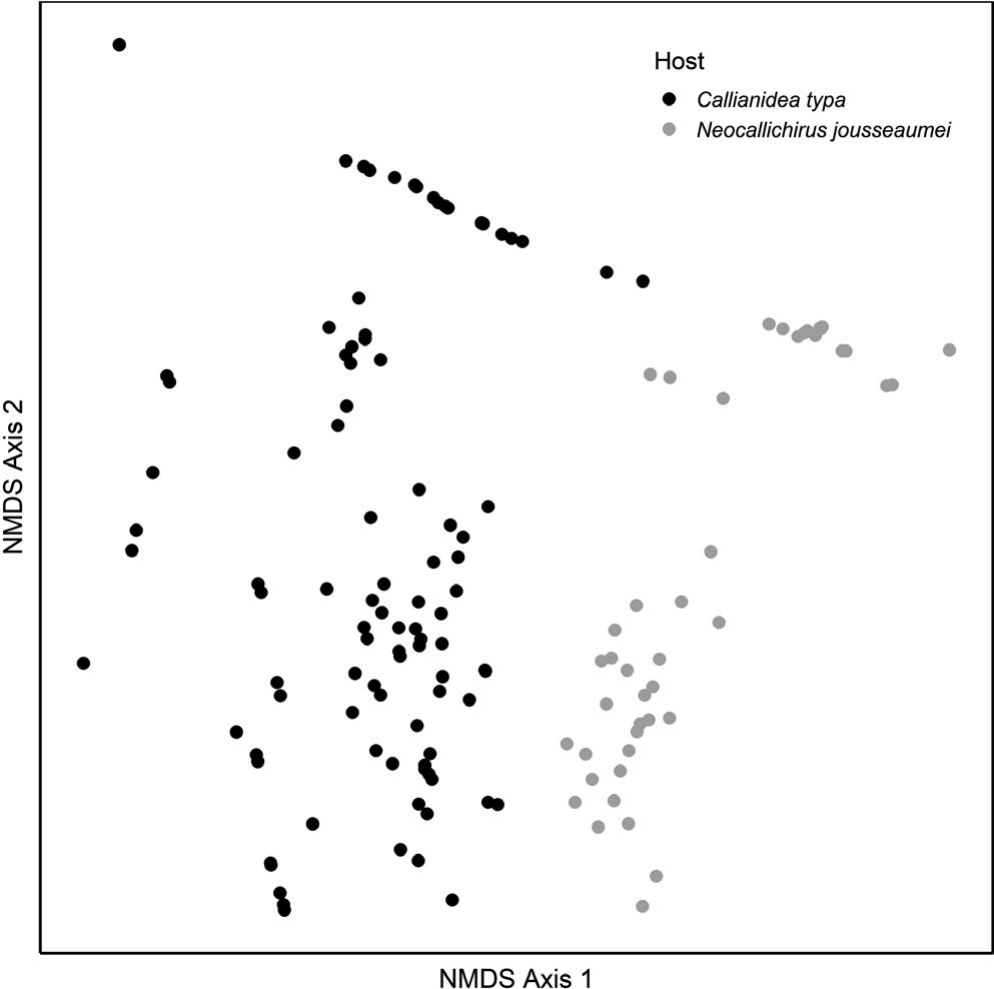
Relationship between symbiotic copepod intensity on the sub‐dominant sites carapace, legs, and abdomen/telson and intensity on the dominant site, chelae, for the host *N. jousseaumei*. There were no significant relationships between dominant and subdominant sites for the host *C. typa*

We discovered two co‐occurrence species of ghost shrimp, *Neocallichirus jousseaumei* (Nobili, 1904) and *Callianidea typa*, Milne Edwards, 1837 that served as hosts to copepod symbionts. The mean body length of *N. jousseaumei* and *C. typa* were 45 and 55 mm, respectively. We found 8,052 individual of *Clausidium persiaensis* Sepahvand & Kihara, 2017 associated to 125 individuals of *C. typa* and 1,685 specimens of *Clausidium iranensis* Sepahvand et al., 2019 on 224 specimens of *N. jousseaumei*. We observed absolute host specificity, that is, *C. persiaensis* was only found on *C. typa*, and *C. iranensis* was only found on *N. jousseaumei*. *Clausidium persianensis* infestation level on *C. typa* was higher than infestation levels of *C. iranensis* on *N. jousseauemei* (mean intensity was 64.41 for *C. typa* and 15.04 for *N. jousseaumei*). Copepod densities on *N. jousseaumei* and *C. typa* were correlated with body size of host shrimps. While the two host species spanned a similar range in size and symbiont density on both host species was low at small host sizes, density increased much more rapidly with host size on *C. typa* to the extent that densities were 4× higher on the largest *C. typa* compared to *N. jousseumei* (Figure 4). We detected a strong pattern of microhabitat occupancy across hosts. The most frequently occupied microhabitat on *C. typa* was the most anterior portion of the dorsal and lateral of carapace surface and gill chamber (Cara) with approximately 85% of symbiotic copepods present at that microhabitat, followed by the chelipeds (Chel, 5%), thoracic legs (legs, 3%), and Abdomen and telson (Abtel, 2%; Figure 5). Copepods were most frequently attached to *N. jousseumei* at the chelipeds (Chel, ca 80%) with the carapace and abdomen/telson microhabitats infrequently occupied (8% and 2%, respectively, Figure 5). For both symbiont species, microsite preference was highly nonrandom. On *C. typa*, intensity on the dominant site, carapace, was strongly predicted by both host sex and host size, though there was no interaction between these factors (Table 1). For the nondominant sites (chelae, legs, and abdomen/telson), host sex was the only significant predictor. However, for all sites, the significant sex effect was largely driven by the inclusion of juveniles whose sex could not be identified (mean symbionts ± *SD*, female = 74.5 ± 38.6, male = 68.3 ± 39.1, juvenile = 9.5 ± 4.2). There was also no significant relationship in intensity between the dominant site, carapace, and nondominant sites (Table 1). For *N. jousseaumei*, intensity on the dominant site, chelae, was significantly related to both host size and host sex, and there was a significant interaction between the predictors (Table 1). For *N. jousseaumei*, the host sex effect was not simply driven by lower intensities on juveniles. There was a significant difference between males and females (one‐way ANOVA, *F* = 18.08, *p* = .00049) with females having nearly twice the intensity of males (20.5 ± 13.3 vs. 10.9 ± 7.2). Intensity of copepods on the dominant site, chelae, was also a significant predictor of intensity at all nondominant sites. Correlations of relative intensity between dominant and nondominant sites were all negative (Figure 6) though the relationship with Abdomen/Telson intensity was not strictly linear because of numerous shared zeros.

**TABLE 1:** Results of linear mixed models for intensity of symbiotic copepods on each host attachment site (carapace, chelae, legs, and abdomen/telson) for the two ghost shrimp host species *C. typa* and *N. jousseaumei*

*Callianidea typa*																
Site	Host sex				Host size				Host sex × Host size				Carapace intensity			
	Num *df*	Den *df*	*F*	*p*	Num *df*	Den *df*	*F*	*p*	Num *df*	Den *df*	*F*	*p*	Num *df*	Den *df*	*F*	*p*
Carapace	2	119	27.7	<0.0001	1	119	69.8	<0.0001	2	119	2.19	0.12	NA	NA	NA	NA
Chelae	2	118	5.15	0.007	1	118	0.55	0.46	2	118	0.13	0.88	1	118	0.14	0.71
Legs	2	118	5.17	0.0071	1	118	0.055	0.46	2	118	0.13	0.87	1	118	0.14	0.71
Ab‐Telson	2	118	3.22	0.043	1	118	1.41	0.23	2	118	0.08	0.93	1	118	0.31	0.58

*Neocallichirus jousseaumei*																
Site	Host sex				Host size				Host sex × Host size				Chelae intensity			
	Num *df*	Den *df*	*F*	*p*	Num *df*	Den *df*	*F*	*p*	Num *df*	Den *df*	*F*	*p*	Num *df*	Den *df*	*F*	*p*
Chelae	2	106	384.7	<.0001	1	106	34.5	<0.0001	2	106	5.0	.008	NA	NA	NA	NA
Carapace	2	105	21.9	<.0001	1	105	12.9	.0005	2	105	0.64	.53	1	105	32.1	<.0001
Legs	2	105	13.2	<.0001	1	105	9.14	.0032	2	105	0.09	.91	2	105	17.8	.0001
Ab‐Telsn	2	105	2.56	.082	1	105	0.40	.53	2	105	1.58	.21	1	105	25.6	<.0001

Note

The variables Host sex, Host size, and their interaction were included in all models. For subdominant sites, that is, sites that did not rank first in copepod abundance, intensity at the dominant site (listed first) was also included in the linear model to examine whether abundance at the dominant site affected intensity at subdominant sites

**FIGURE 7:**
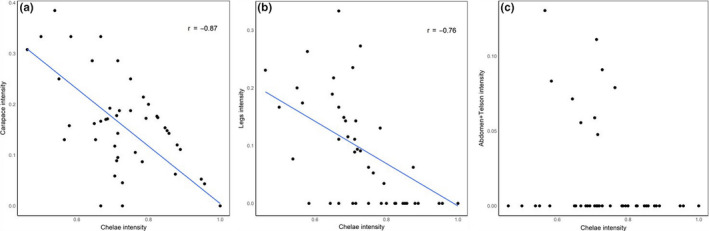
Nonmetric multidimensional scaling ordination of symbiotic copepod distributions on the two ghost shrimp host species, *C. typa* and *N. jousseaumei*

The multivariate distribution of symbiotic copepods clearly differed between hosts (Figure 7). The ordination was excellent in two dimensions with a Stress of 0.073. The two host species differed most prominently along NMDS Axis 1, an axis strongly related to chelae in the positive direction and carapace in the negative direction (Table 2). PERMANOVA showed strong effects of host species, host size, and host sex, as well as all 2‐way interactions (Table 3). There was no significant difference in multivariate dispersion between the 2 hosts (*p* = .084).

**TABLE 2:** Correlations of site variables to NMDS ordination axes

	Axis 1	Axis 2
Chelipeds	0.47	−0.20
Carapace	−0.63	−0.01
Legs	−0.25	−0.80
Abdomen‐Telson	−0.29	−0.28

**TABLE 3:** Results of PERMANOVA testing the effects of host species, host size, and host sex on the multivariate distribution of symbiotic copepods on ghost shrimp hosts

Factor	*df*	Sums of squares	*F*	*r* ^2^	*p*
Host species	1	32.9	502.8	.54	<0.001
Host size	1	4.0	60.6	.065	<.001
Host sex	2	1.9	14.7	.32	<.001
Species × size	1	4.7	71.8	.078	<.001
Species × sex	2	1.6	12.0	.026	<.001
Size × sex	2	0.8	5.9	013	<.001
Species × size × sex	2	0.3	2.12	.0047	.067
Residuals	225	14.7		.24	

## 4 DISCUSSION

Both species of symbiotic copepods displayed extremely high host specificity. Among four species of callianassid ghost shrimps that live in the intertidal zones of the Persian Gulf, *Clausidium iranensis* Sepahvand et al., 2019 and *Clausidium persiaensis* Sepahvand & Kihara, 2017, colonized only *Neocallichrus jousseaumei* (Nobili, 1904) and *Callianidea typa* Milne Edwards, 1837, respectively. Although more intensive sampling could potentially yield symbionts of other hosts, our results do suggest high host specificity within clausidium copeods assemblage may relate to host physiology, ecology, or behavior host of the clausidium copepopds. Other factors, for example, ecological or ethological conditions, may account for the failure of the clausidium to occupy the other ghost shrimps species. The ghost shrimps are different in habitat selection, trophic modes as well as in burrowing pattern (Griffis & Suchanek, 1991; Sepahvand, Sari, Salehi, Nabavi, & Ghorbanzadeh, 2013). Hence, each of these factors may affect the host selection of clausidium copepods in the Persian Gulf. Host choices may reflect both contemporary selection due to different costs and benefits of burrowing shrimps in different locations, and the phylogenetic host specificity of any mutual selection which has occurred between participants. Thus, while clausidium copepods are clearly adapted to associated lifestyle with ghost shrimps both behaviorally and morphologically, it is not known whether this adaptation is tailored to particular shrimp species.

The data analysis showed that host‐size adjusted density of clausidium copepods was affected by the host species and the host sex. While the two species of ghost shrimp hosts are similar in size, the symbiont density × host size relationship differed markedly between hosts. While both hosts had few symbionts at sizes < 35 mm, the density of symbionts on *C. typa* increased far more rapidly with host size than on *N.jousseaumei*. Symbiont densities on the largest sized *C. typa* were 4× greater than comparably sized *N. jousseaumei*. While the explanation for this disparity is not readily apparent, one explanation is that the symbiotic copepod *C. persiaensis* preferentially attaches to *C. typa*, or that *N. jousseaumei* exhibits selective host defense and repels the symbiont, possibly through removal by grooming behavior.

Another possibility is that the feeding mechanism of *C. typa* and *N. jousseaumei* may influence the abundance of copepods. Ghost shrimp feed in a variety of ways including filtration of plankton and deposit feeding, and generally consume microalgae and other diatoms (Felder & Griffis, 1994). Most callianassid ghost shrimp feed by sifting sand for microscopic organisms using their mouthpart to remove food particles from setae of the maxillipeds (Pohl, 1946). Hayes (1976), showed that recruitment of the planktonic host‐seeking stage of copepods, Copepodid I in clausidium copepods may depend on the host's feeding mechanism, hence the trophic mode of ghost shrimp may affect the symbiont copepods in host preference process.

We also found that there was a significant difference in the number of copepods selecting each host sex. This result could signify that copepods base their selection of host in a hierarchical fashion, with host species forming a first hierarchy, and host size a second. Since females are generally larger in size than male higher copepod colonization on female specimens may be a result of size and not sex.

Explanations for host selection may also be evolutionary in origin and be related to contact time among these copepods and ghost shrimps in the Persian Gulf. The Persian Gulf is relatively young with coastlines that formed only in the past 3,000–6,000 years (Riegl & Purkis, 2012) and the evolutionary process within it may have been affected by historical events such as glaciation and sea level fluctuation (Sheppard et al., 2010). Regarding the short history of the region, contact time between ghost shrimps and *Clausidium* copepods has been limited, as has the time during which clausidium copepods have adopted new species of ghost shrimp hosts. Although some studies suggest that coevolution between symbiont and host can occur rapidly when parasitism is high (Soler, Martinez, Soler, & Møller, 1994; Takasu, Kawasaki, Nakamura, Cohen, & Shigesada, 1993), it is not clear whether the intensity of selection, either positive or negative, and time since the clausidium copepod introduction have been sufficient for ghost shrimps to have evolved responses.

Each symbiont species displayed strong patterns of microhabitat selection. This site selection was apparent in two ways: (a) within a single symbiont species, there was clear site selection on a host; (b) there were also strong differences in site selection between the two hosts. *Clausidium persiaensis*, was only found on *C. typa* and almost exclusively on the carapace and within the gill chamber, while *C. iranensis* was only found on *N. jousseaumei* and showed extremely strong preferences for the chelae and anterior walking legs.

Our result showed that for both symbiont species, microsite preference was highly nonrandom. Possible explanations for site preferences are minimizing risks from host defensive grooming behaviors, or constraint from environmental parameters such as current strength.

We consider three hypotheses that may explain microhabitat preference in two clausidium copepod species on their hosts: first, grooming behavior (GB) of the ghost shrimp (Bauer, 1981) and copepods occupying protected zones making them inaccessible to " grooming "; second, burrowing behavior (BB) of hosts determines the current velocity experienced by symbionts (Dworschak, Felder, & Tudge, 2012) and copepods choose microhabitats that minimize the force of "flow"; third, niche partitioning (NP) could be a strategy to increase the possibility of finding mates. Diverse grooming structures and behaviors have evolved in decapod crustaceans in response to the selective pressure of fouling (Bauer, 1981). General body grooming of decapods, performed by serrate setal brushes on chelipedes and/ or posterior pereiopods (Bauer, 1981). Fifth pereiopods as a main appendage for grooming of the carapace and gills of *C. typa* and *N.jousseaumei* are almost morphologically similar, while these shrimps are different in maxiliped 3 and in another appendages (major and minor chelipeds, pereiopods 2–4).

The (BB) and the (NP) hypotheses explain the microhabitat selection advantage in *Clausidium*.

Two ghost shrimp hosts do differ in behavior in several ways including their patterns of burrowing, and habitat selection (Griffis & Suchanek, 1991). Burrowing patterns determine the current velocity of water in burrows and consequently on the body of the ghost shrimps (Griffis & Suchanek, 1991). Possible differences in water current strength in the burrow of hosts may also explain the differences of microhabitat preferences in clausidium copepods. The flattened body shape of the genus *Clausidium* (Figures 1b, 2b) is probably an adaptation against stress experienced at their habitats. Marin and Nascimento (1993) analyzed the body size and habitat of ghost shrimp (*Callichirus garthi* Retamal, 1975) as factors affecting the distribution, abundance, and fecundity of *Clausidium* spp. The results of that study suggested that the density of *Clausidium* spp. per host was dependent upon host's habitat type and recruitment. Most specimens collected in this study were found coupling during the mating process (Figures 1b, 2b), providing evidence that mating may benefit from microsite partitioning on the host. However, Timi (2003) suggested that microhabitat restriction of *Lernanthropus cynoscicola* (parasitic copepod) is not due to facilitation of mating. Additionally, he showed that aggregation among individuals of the same sex was stronger than among males and females, and the co‐occurrence of both sexes did not depart from that expected by chance. While this present study was not designed to explore the ecological significance of host site preference, we suggest that some evidence does support the burrowing and mating hypotheses. However, the discussed hypotheses should be further studied to illuminate the evolutionary and adaptive advantages of niche differentiation in copepods and their hosts.

Rohde (1979) reviewed intrinsic and extrinsic factors those are responsible for niche restriction in parasites. The author emphasized that intrinsic (intraspecific) factors are largely responsible for niche restriction. Rohde (1979) argued that intrinsic factors play some roles in determining niches in monogen species and suggested that narrow microhabitats may function to enhance mate‐locating chances. On the other hand, site selection within the host also may relate to the physicochemical environment (e.g., Bychowsky, 1961; Wootten, 1974).

Positive correlations between host size and symbiont density or biomass are frequently reported, especially in parasite systems (e.g., Arneberg, Skorping, Grenfell, & Read, 1998; Grutter & Poulin, 1998; Mohr, 1961; Poulin, 2007; Saad‐Fares & Combes, 1992). However, some authors have also observed that lower levels of parasitism may occur in the largest hosts (Shotter, 1973; Kabata, 1959; Etchegoin & Sardella, 1990). Our result revealed that *N. jousseaumei*, despite a larger body size, was host to fewer copepods on average (mean 15.4) when compared to *C.typa* (mean 64.41). These results do not agree with a previous study in which the abundance of clausidium copepods was directly related to the size of host (Marin & Nascimento, 1993). It is possible that the difference in densities level of clausidium on the body of hosts relate to the host's physiology, since member of the *Clausidium* genus is same in biology and attachments mechanisms. Corsetti and Strasser (2003) showed that copepod densities are correlated to the host species and host size, but host sex was unimportant.

In conclusion, there is strong evidence from field surveys that *Clausidium iraniensis* prefers the large chelipeds of *N. jousseaumei*, and *C. persiaensis* primarily occupies the gill chamber of *Callianidea typa*. Unfortunately, both the proximate and ultimate causes of this preference are difficult to ascertain because the exact nature of the cost/benefit relationship between clausidium copepods and their hosts is unclear. Future research should focus on quantifying the mutual costs and benefits to both clausidium copepods and their ghost shrimp hosts, and examining the relationship in the context of changing species and environmental conditions.

## CONFLICT OF INTEREST

The authors declare that they have no conflict of interest.

## AUTHOR CONTRIBUTIONS

**Vahid Sepahvand:** Conceptualization (lead); data curation (lead); formal analysis (equal); methodology (equal); project administration (lead); software (equal); writing – original draft (lead). **Bryan L. Brown:** Formal analysis (lead); resources (equal); software (lead); supervision (equal); validation (equal); visualization (equal). **Ali Gholamifard:** Project administration (equal); visualization (lead); writing – review and editing (equal).

## Data Availability

The raw data are available at Dryad with this https://doi.org/10.5061/dryad.4qrfj6q6p.
